# Trauma-informed healthcare from the perspectives of women who have experienced sexual violence in adulthood: a systematic review and meta-ethnography

**DOI:** 10.1186/s12913-025-13584-x

**Published:** 2025-11-27

**Authors:** Síofra Peeren, Elsa Montgomery, Angela Sweeney, Gilda Spaducci, Anjuli Kaul, Demelza Smeeth, Sian Oram

**Affiliations:** 1 King’s Women’s Mental Health, Department of Health Services and Population Research, Institute of Psychiatry, Psychology and Neuroscience, King’s College London, London, UK; 2 Service User Research Enterprise, Department of Health Services and Population Research, Institute of Psychiatry, Psychology and Neuroscience, King’s College London, London, UK; 3 Methodologies Division, Florence Nightingale Faculty of Nursing, Midwifery & Palliative Care, King’s College London, London, UK; 4 Lambeth Health Determinants Research Collaboration, Department of Public Health, Lambeth Council, London, UK

**Keywords:** Meta-ethnography, Systematic review, Qualitative, Survivor research, Trauma-informed approaches, Healthcare, Sexual violence

## Abstract

**Background:**

Sexual violence is a global public health problem with wide-ranging and long-term health impacts. Survivors may experience further harm when healthcare settings replicate dynamics of sexual violence (re-traumatisation), underscoring the need to understand survivor perspectives on how healthcare settings can prevent harm and promote healing (trauma-informed approaches). We synthesised qualitative research exploring healthcare experiences and expectations among female survivors of sexual violence in adulthood.

**Methods:**

Systematic review and meta-ethnography. We searched 14 electronic databases (upper date limit 13th February 2024), supplemented by citation tracking, to identify qualitative studies exploring healthcare experiences and expectations among female survivors of adulthood sexual violence. First- and second-order constructs (participant quotes and author interpretations) were extracted and synthesised using meta-ethnography, including reciprocal and refutational translations and a line of argument synthesis. Quality appraisal was conducted using the CASP and COREQ tools, alongside additional criteria to document trauma-sensitive research considerations. Confidence in review findings was assessed using CERQual.

**Results:**

Fifty studies were synthesised, generating a conceptual model and line-of-argument synthesis positioning demonstrating trustworthiness as central to trauma-informed healthcare. Trust was built by countering key harms of sexual violence, illustrated in three themes. The first theme, *name the violence*, described how survivors needed time, safety, and reassurance to recognise and make sense of experiences of sexual violence, which was often hidden or minimised, to reduce self-blame and shame and begin to heal. The second theme, *make sexual violence visible*, explored how providers and services could challenge silencing by asking, believing, responding, and framing sexual violence as a human rights violation. The third theme, *bear witness*, emphasised the importance of affirming survivors’ dignity, agency, and autonomy to counter the dehumanising effects of sexual violence.

**Conclusions:**

Healthcare can replicate the harms of sexual violence, but also holds unique potential to support healing when survivors are met with validation, affirmation, dignity, and respect. Demonstrating trustworthiness is central to this process and depends not only on individual provider actions but also on system-level conditions that shape safety, visibility, and equity. System-wide trauma-informed approaches are needed to build trust and address systemic conditions and practices that perpetuate harm and undermine healing.

**Supplementary Information:**

The online version contains supplementary material available at 10.1186/s12913-025-13584-x.

## Background

Sexual violence is a global public health problem and a significant and gendered determinant of health. [1]. Defined as “any sexual act … against a person’s sexuality using coercion,” it is a violation of human rights that is rooted in, and perpetuates, gender inequality [2]. Globally, a third of women report intimate partner violence (IPV) or sexual violence in adulthood [3] and in the UK, almost a quarter (23%) of females report sexual assault or rape since age 16 [4]. Among women, sexual violence is commonly perpetrated by someone known to a survivor [5] and therefore may be intertwined with other harms such as betrayal and psychological abuse [6, 7]. It also frequently co-occurs with other forms of gender-based violence, such as intimate partner violence and stalking [5] and is often experienced repeatedly over a lifetime, including in childhood [5]. These intersecting and cumulative experiences contribute to a compounded burden that leads to more severe and wide-ranging health consequences, most often reproductive, mental health, and social well-being impacts [2, 8], as well as higher rates of healthcare use [9, 10]. Yet, experiences of sexual violence often remain hidden due to shame and societal silencing [11], preventing access to timely and tailored support [12].

Healthcare providers, services and systems play a critical role in mitigating harm after sexual violence through early identification and response (secondary prevention), and by supporting long-term healing and reducing lasting health impacts (tertiary prevention) [12]. However, because broader social structures shape healthcare systems, they can actively reflect and reproduce inequity and power dynamics [13–15] - an important consideration given that sexual violence is also rooted in gender inequality and shaped by broader social inequity [2]. For example, victim-blaming attitudes, which are shaped by gendered and patriarchal ideas [16], can lead to responses to disclosure that silence survivors and discourage further help-seeking [17]. Similarly, the societal silencing that surrounds sexual violence [11] can be mirrored in healthcare environments that fail to acknowledge its prevalence or are ill-prepared to respond, particularly in settings that may see a high number of survivors [11, 18, 19]. Routine practices within healthcare can also reproduce power imbalances or experiences of violation and powerlessness similar to sexual violence, including coercion [11], de-legitimising service user perspectives [11, 19], or crossing body boundaries [20]. These experiences of violation or coercion in healthcare are often gendered, rooted in similar societal ideas about women and female bodies that excuse or minimise sexual violence [21], such as assumptions of irrationality, passivity, or diminished need for consent [22, 23].

Despite its importance, the health system response to sexual violence remains inconsistently supported in policies and budgets, including provider training and development and implementation of sexual violence-specific protocols, policies, and guidelines [12]. This is despite evidence that the economic costs of sexual violence are considerable and borne mainly by public services such as health systems [24, 25]. These costs reflect the long-term and multifaceted impacts of sexual violence on health and well-being [2] - impacts that are likely to be exacerbated when survivors do not receive timely and appropriate care.

Trauma-informed approaches are an important part of strengthening health system responses to sexual violence [26]. Trauma-informed approaches aim to transform systems by recognising that harm can be embedded in dominant practices, policies, and power structures [11]. These approaches work to dismantle such harm by fostering organisational environments grounded in principles like power-sharing, trust-building, and relational safety, aimed at actively countering the dynamics of violence and promoting healing [27, 28]. Interest in trauma-informed approaches in healthcare systems is increasing; for instance, a recent research priority setting exercise on sexual violence identified a need for research to explore how healthcare services can become more trauma-informed [26]. Crucially, though, research into the development and implementation of trauma-informed approaches must centre survivors’ perspectives and priorities, recognising that survivors best understand what supports healing and what risks further harm [29].

Although the World Health Organisation (WHO) called for a strengthening of the health system’s response to violence against women a decade ago [12], research synthesising sexual violence survivors’ perspectives on trauma-informed healthcare remains scarce. Most qualitative reviews focus on IPV and do not address the specific needs of sexual violence survivors – including those who have experienced sexual violence from a partner [30]. While many elements of a trauma-informed response are similar across different forms of violence against women, sexual violence is described by survivors to have a unique context and impact [7], indicating that sexual violence survivors could benefit from a tailored response [31].

By synthesising qualitative research exploring survivors’ experiences and expectations of healthcare, our systematic review addresses these knowledge gaps. Recognising the importance of placing survivors’ perspectives at the heart of research that affects them, our review was survivor-led (the first and third authors identify as trauma survivor researchers) and was guided by survivor-generated ethical frameworks and epistemology [32, 33]. We chose to conduct a meta-ethnography because it is well suited to generating new understandings of complex social and public health challenges such as sexual violence while preserving the context of the studies [34–37]. Additionally, its focus on developing new conceptual insights allowed us to develop a conceptual model, grounded in survivors’ perspectives, which can be used to inform future policy, practice, and research on trauma-informed approaches. Our review was guided by the following question: What are female adulthood sexual violence survivors’ experiences and expectations of healthcare?

## Methods

This paper follows eMERGe reporting guidance for meta-ethnographies [34] and a checklist is provided in Appendix A. The protocol was registered on PROSPERO on 14/01/2019 (reference: CRD42019120101).

### Search strategy and search processes

Comprehensive searches were conducted because we aimed to identify all qualitative studies that were relevant to the review question. SP tested and refined search terms in Ovid (Appendix B) and adapted them for other platforms. The searches included medical subject headings (MeSH) and keywords using truncation (*) within title or abstract fields and used Boolean terms “OR” and “AND” to combine searches within and between categories. SP searched fourteen electronic databases on the 22^nd of^ July 2019 and again to update searches on the 13^th of^ February 2024 with no lower time limit. The following databases were searched: CINAHL, EMBASE, HMIC, BNID, ASSIA, IBSS, SSCI, MEDLINE, PsycINFO, MIDIRS, OATD, NDLTD, OpenGrey and SCIE Online. SP and AK supplemented the searches through forward and backwards citation searching in Web of Science and Google Scholar.

### Inclusion/exclusion criteria

Studies were screened against the following inclusion criteria: (1) primary qualitative research (or mixed methods research where qualitative findings could be separately extracted); (2) included participants described as female (or women, if sex was not reported) and as having experienced sexual violence in adulthood (defined as age 16 or older at the time of the violence); (3) published in peer-reviewed or grey literature, including postgraduate research theses/dissertations, book chapters or reports; (4) explored experiences/expectations of healthcare; (5) written in English or Dutch. We excluded all other study designs.

While recognising that sexual violence can affect people of all genders, this review focuses on survivors described in primary studies as female (or women, if sex was not reported). This decision reflects the sex-specific nature of healthcare, such as maternity and gynaecology – settings which also have distinct and unique implications for sexual violence survivors [20, 22]. We aimed to illuminate how health care experiences are influenced by the intersection of sexual violence with male-dominated medical systems and the historical marginalisation of female bodies in healthcare [38, 39], as well as the ways both sexual violence and healthcare are shaped by gendered norms and broader gender inequalities [21, 23].

Studies that included survivors of different types of violence were included if data could be separately extracted for participants who met the other inclusion criteria. Corresponding authors were contacted by SP for assistance where needed. If a study’s findings were published both in a thesis and a journal, the report with the richest and most detailed information was included. We attempted to access full texts using multiple approaches, including submitting library and interlibrary loan requests and contacting authors directly where contact information was available.

### Selecting primary studies

SP imported abstracts into Cadima software [40] and removed duplicates using the software. Titles and abstracts were screened by SP, DS and AK against the inclusion and exclusion criteria. GS independently screened 250 abstracts with 90% agreement. Disagreements were resolved through discussion until consensus was reached between the two reviewers on all full-text articles. A third reviewer (SO) was available to adjudicate if consensus could not be reached, but this was not required. SP screened all 504 full-text articles, of which 25 were independently screened by GS (approximately 5%). Agreement was 80% and again disagreements were resolved through discussion until we reached 100% agreement. SP double screened all papers identified by AK through forward and backward citation searching.

### Approach to analysis

Our approach to meta-ethnography was adapted from Noblit and Hare [36]. It was guided by several worked examples [41–45] and a methodological systematic review providing guidance on the complex synthesis and translation phases of meta-ethnography [35].

**Reading and data extraction approach.** SP read each report several times and extracted the following study information in an Excel sheet: publication year; participant characteristics; contextual information such as policy context, aims, and epistemology; approach to analysis; key themes; participant quotes; and author interpretations. GS extracted data for 5 studies (approximately 10% of those included) and inconsistencies were resolved through discussion. Drawing on Schütz’s [46] concept of first and second-order constructs, we extracted both participants’ quotes (first-order constructs) and author interpretations (second-order constructs), taking a similar approach to other meta-ethnographies in health research [35, 42, 44]. We analysed first and second-order constructs together to recognise that participant quotes are chosen by authors to illustrate the authors’ interpretations of what participants said [35, 47].

**Assessing the quality of included studies.** Quality assessment is contested in qualitative synthesis. Published qualitative research often contains limited detail on methodology due to word limits and therefore a low score on quality assessment does not mean that the findings are unreliable [48–50]. We followed Noblit and Hare’s [36] argument to include all relevant studies irrespective of quality assessment scores, but we decided that it was important to assess the strength of the evidence given our aim to inform research, policy, and practice [47, 49]. We completed the COREQ statement [51] to facilitate a close reading of the texts and used the Critical Appraisal Skills Programme Qualitative Checklist (CASP-QC) [52] to document our quality assessment process. We supplemented the CASP-QC with three additional quality criteria that illustrated aspects of research quality that are important to survivors [53, 54]. These were: (1) do the authors report trauma-informed ethical considerations? (2) do the authors report survivor involvement?; (3) do the authors acknowledge and/or address power imbalances? (see Appendix C for the CASP and additional criteria).

**Assessing the quality of the review findings.** We used the CERQual [55] to assess each finding for methodological limitations; coherence; adequacy; and relevance. To document methodological limitations, we used the CASP tool to categorise studies under the following headings: ‘no or minor concerns,’ ‘minor concerns,’ ‘moderate concerns’ or ‘serious concerns.’ Each sub-theme was rated with a confidence level of either high, moderate, low, or very low in the CERQual evidence profile (Appendix D).

**Process for determining how studies are related.** We compared key findings, methodologies, and contexts across reports to understand relationships between studies. To manage the large amount of data, we first sorted the first and second-order constructs into broad, data-driven categories, e.g., ‘communication.’ We then used matrix queries in NVivo to identify intersections between studies in terms of their findings, methodologies, and contexts.

**Process of translating studies.** We used a process of translation to compare and synthesise the findings of included studies [36]. This involved identifying key concepts, findings, and themes in each study, and then systematically comparing them with those from other studies to understand and express how they related, overlapped, or differed. Studies were compared in chronological order. This process was documented in an Excel matrix, with each study summarised in a dedicated cell capturing its key concepts, findings, and relevant contextual details such as methodological approach. This preserved the context and nuance of the original studies and left a clear audit trail. Appendix E outlines an example of how we produced a translation from different findings.

**Synthesis process.** We considered the translations as a whole [36] to generate third-order constructs which are reported here as themes and sub-themes. We conducted three syntheses: a reciprocal translation, where we focused on the similarities between studies; a refutational translation, where we examined inconsistencies between studies and paid attention to each study’s underlying assumptions, motivations, and epistemology [36, 37]; and a line of argument synthesis, where we interpreted the relationship between the third order constructs and highlighted a key but hidden concept that connected the reports [36]. An example of how we moved from translations to third-order constructs is provided in Appendix E. All authors contributed to discussing and refining the themes and sub-themes to ensure rigour and accurate representation of the data.

**Reflexivity and positionality**. Reflexivity is essential to both survivor research [56] and meta-ethnography [49]. To facilitate an ongoing process of reflexivity, the first author kept a reflective notebook, engaged in regular discussions with the second, third, and final authors, and attended monthly group reflective research supervisions led by a clinical psychologist. SP is a White female researcher with lived experience related to the review topic; her research focuses on improving health and social care responses to trauma, violence, and abuse, and on developing methodologies for research led and shaped by people with lived experience. EM is a White female midwife by background; her research focuses on trauma-informed care for survivors of child sexual abuse. AS is a White, female survivor researcher with lived experience connected to the review topic; her research centres on the experiential knowledge of survivors. SO is a White female applied health researcher; her research focuses on interpersonal trauma, its intersection with sex and gender and institutional and societal structure, and its relationship to mental health. All authors believe that healthcare systems have a responsibility to address sexual violence as a form of gender-based violence and that survivors know best what heals and what harms. We also believe that research should challenge ingrained cultural beliefs that can de-legitimise survivors’ perspectives, including, “who has the power to define and determine ‘illness’ or ‘disorder’ and how it should be treated” [57].

## Results

### Outcome of study selection

**Fig. 1:**
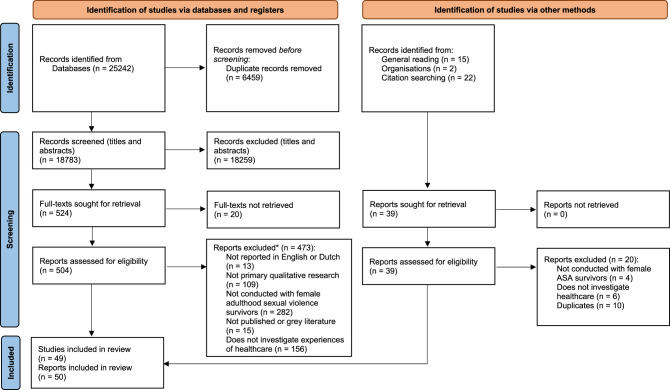
PRISMA 2020 flow diagram illustrating the study selection process

The PRISMA 2020 statement [58] is presented in Fig. 1. At least 606 survivors of adulthood sexual violence were included in the analysis.

### Study characteristics

Table 1 describes the characteristics of the included studies. Many survivors reported multiple experiences of sexual violence from multiple perpetrators at different times in their lives and often co-occurring with other forms of violence and abuse. Most studies (*n* = 41) were conducted in high-income countries, primarily the USA and UK, whereas nine studies were conducted in low- and middle-income countries [59–67]. Healthcare settings included specialist sexual violence (*n* = 14) [60–62, 68–78]; mental health (*n* = 9) [59, 64, 79–85]; sexual, reproductive and maternal health (*n* = 10) [59, 66, 67, 69, 86–91]; Veteran’s Health Administration (VHA) (*n* = 3) [92–94]; emergency or hospital care (*n* = 4) [95–98]; and dentistry (*n* = 2) [99, 100]. Some studies explored general healthcare experiences or help-seeking of which healthcare was one aspect (*n* = 10) [63, 65, 101–108].

**Table 1:** Study characteristics

Study	Participants (included)	Health care setting	Marginalised survivors	Data collection	Theory/methodology/approach to analysis	Country	Participants
Abrahams (2017) [59]	43 (14)	Mental health and HIV		In-depth interviews	Framework approach to thematic analysis	South Africa	Mixed (all SV)**
Ahrens (2002) [68]	8 (5)	Specialist SV		Interviews	Feminist lens; codebook approach	USA	ASA
Alyce (2022) [99]	17 (7)	Dentistry		Semi-structured interviews	Survivor research epistemology; thematic analysis	UK	CSA**
Barros et al. (2015) [60]	11	Specialist SV		Semi-structured interviews	Content analysis	Brazil	ASA
Batistetti et al. (2020) [61]	11	Specialist SV		Semi-structured interviews	Content analysis	Brazil	ASA
Birdi (2022) [69]	10 (6)	Sexual health and specialist SV		Semi-structured interviews	IPA	UK	Mixed (all SV)**
Birthrights and Birth Companions (2019) [86]	12 (NR)	Maternity/perinatal	Multiple disadvantage	Semi-structured interviews	Human rights and trauma-informed lens	UK	Mixed**
Campbell et al. (2013) [70]	20 (11)	Specialist SV		In-depth qualitative interviews	Inductive approach	USA	Mixed (all SV)**
DeLoveh (2017) [101]	14 (13)	General		Semi-structured interviews	Grounded Theory	USA	ASA*
Draucker (1999) [79]	33	Mental health		Interviews	Content analysis	USA	ASA
Du Mont et al. (2009) [71]	19	Specialist SV		Semi-structured interviews	Content analysis	Canada	ASA
Ericksen et al. (2002) [72]	8	Specialist SV		Semi-structured interviews	Interpretive epistemology; latent content analysis	Canada	ASA
Fehler-Cabral et al. (2011) [73]	20	Specialist SV		Semi-structured interviews	Open coding followed by pattern coding	USA	ASA
Fish and Hatton (2017) [80]	16 (5)	Mental health	Women with intellectual disabilities	Interviews and ethnographic fieldwork	Ethnography; feminist and disabilities studies analysis	UK	Mixed**
Guerette (2007) [102]	12 (6)	General		Structured interviews	Both inductive and deductive coding	USA	ASA
Gutzmer et al. (2016) [103]	19	General	African American women	Semi-structured interviews	Informed by theories about the sexual division of power; codebook approach	USA	ASA
Halvorsen et al. (2013) [87]	10	Maternity/perinatal		Semi-structured interviews	Text was divided into meaning units that were coded.	Norway	ASA
Hellman (2016) [104]	9 (2)	General		In-depth, semi-structured interviews	Heideggerian hermeneutic phenomenology	USA	ASA
Holton (2016) [62]	10 (7)	Specialist SV		In-depth interviews	A thematic index was created during familiarisation	South Africa	ASA
Hutschemaekers et al. (2019) [74]	12	Specialist SV		Semi-structured interviews	Open coding led to the development of a coding framework	Netherlands	ASA
Isaac (2024) [81]	29	Mental health		In-depth, unstructured interviews	Braun and Clarke’s reflexive thematic analysis	Australia	ASA
Jacobs (2016) [92]	16	VHA		Unstructured interviews	Heideggerian hermeneutic approach	USA	MST
Jonsdottir (2022) [88]	9 (3)	Maternity/perinatal		1–2 in-depth interviews	Blend of phenomenology, hermeneutics and constructivism	Iceland	CSA**
Jordan (2001) [75]	48 (34)	Specialist SV		In-depth interviews	Not specified	New Zealand	ASA
Kehle-Forbes et al. (2017) [94]	37	VHA		Semi-structured interviews via telephone	Modified grounded theory approach	USA	MST
Kelly (2004) [105]	17 (4)	General	Latina women	Hermeneutic open-ended interviews	Feminism and phenomenology	USA	IPV**
Lissmann (2023) [82]	14 (7)	Maternity and mental health		In-depth interviews	Intersectional feminist approach	UK	Mixed (all SV)**
Monteith et al. (2020) [93]	50 (32)	VHA		Semi-structured interviews	Phenomenological approach to thematic analysis	USA	MST**
Muganyizi et al. (2011) [63]	30 (10)	General		In-depth interviews	Grounded theory informed by Heidegger	Tanzania	Mixed (all SV)**
Naved (2009) [64]	30 (NR)	Mental health		In-depth interviews (Mixed Methods)	A code list was prepared and modified as needed	Bangladesh	IPV**
Nichols et al. (2018) [83]	27	Mental health	Mental health diagnosis	Semi-structured interviews	Intersectionality theory and thematic analysis	USA	ASA
Nobels (2023) [106]	15 (1)	General	Older adults	Semi-structured interviews	Antiageist perspective; realist thematic analysis.	Belgium	Mixed (all SV)**
Olive (2017) [95]	6 (2)	Emergency Care		Semi-structured interviews	Critical realism and postmodern complexity theory	UK	IPV**
Perry et al. (2015) [89]	9	Abortion		In-depth interviews	Content analysis using a codebook	USA	ASA
Place et al. (2019) [65]	23 (13)	General		In-depth interviews	Not specified	Guatamala	Mixed (all SV)**
Porter (2021) [90]	8 (5)	Maternity/perinatal		Semi-structured interviews	Grounded Theory	USA	Mixed (all SV)**
Reisenhofer (2013) [96]	6 (5)	Emergency Care		Semi-structured interviews	Symbolic interactionism and Foucault	Australia	IPV**
Ruiz (2023) [98]	30	Acute care or hospital setting after SV	African American women	In-depth, semi-structured interviews	Critical ethnography; feminism and intersectionality theory	USA	ASA
Santos (2015) [66]	10	Abortion		In-depth interviews	Informed by Marxist philosophy	Brazil	ASA
Sigurdardottir et al. (2018) [107]	1	General		Several in-depth interviews	Phenomenology and Clarke’s situational analysis	Iceland	ASA
Sobel et al. (2018) [91]	30 (NR)	Maternity/perinatal		Semi-structured interviews	Thematic analysis	USA	Mixed (all SV)**
Starzynski et al. (2017) [84]	15 (6)	Mental health		Semi-structured interviews	Ecological theory	USA	Mixed (all SV)**
Ullman (2020) [85]	18	Mental health	African American women	Mixed methods	A process similar to thematic analysis	USA	ASA
Wadsworth (2015) [76]	22	Specialist SV		Semistructured interviews	Feminist theory and constructivism	USA	ASA
Wadsworth (2019) [108]	22	General		Semi-structured interviews	Feminist theory and constructivism	USA	ASA
Walker (2020) [77]	7	Specialist SV		Semi-structured interviews	Thematic analysis with realist epistemology	UK	ASA
Washington (2001) [97]	12 (10)	General including acute hospital care	Black women	In-depth, semi-structured interviews	Black feminist perspective using constant comparison	USA	Mixed (all SV)**
Watt et al. (2017) [67]	15 (14)	HIV	Xhosa women	Semi-structured interviews	Narrative memos and constant comparison	South Africa	ASA
Wolf (2021) [100]	13 (5)	Dentistry		Interviews	Qualitative content analysis	Sweden	Mixed (all SV)**
Wright (2024) [78]	5	Specialist SV		Semi-structured interviews	IPA and feminist lens	USA	ASA

NR = Not reported; CSA = Childhood Sexual Abuse; SV = Sexual Violence; VHA = Veteran’s Health Administration; ASA = Adulthood Sexual Violence and/or Abuse (≥16 years); MST = Millitary Sexual Trauma; IPV = Intimate Partner Violence; NR = Not reported; IPA = Interprative Phenomenological Analysis

*90%+ of the sample met the inclusion criteria

**Quotes which were from female ASA survivors could be separately identified and extracted due to either information found in the paper or through information obtained from the corresponding author

Many studies did not report any theoretical orientation, but 16 studies either directly reported or appeared to draw on theories about oppression, power and/or intersectionality [66, 68, 76, 78, 80, 82, 83, 86, 96–99, 103, 105, 106, 108]. A notable limitation of the literature was that almost all studies (96%) reported no survivor involvement in shaping research questions, methods, or interpretation of data. As a result, most studies were unlikely to have been meaningfully informed by survivor expertise. Exceptions included one study [99] where the lead researcher was a survivor of child sexual abuse and had also consulted survivors to inform her study, and another [86] where women with lived experience of multiple disadvantage were part of a steering group that shaped the study’s findings. No study reported including trans or non-binary survivors, and sex, gender, and gender identity were rarely distinguished by authors. In this review, we use “women” and “survivors” to reflect the language used in the primary studies.

Ten studies focused on the experiences of survivors experiencing marginalisation, including women facing multiple disadvantage [86], women with intellectual disabilities living on locked wards [80], women who had been given a mental health and/or ADHD diagnosis [83], African American women [85, 98, 103], Black women [48], Latina women [26], Xhosa women [49], and older adults [32].

### Outcome of relating studies

All studies contributed to an overall understanding of survivors’ experiences and expectations of healthcare. Some studies were dissimilar but related as inconsistencies between them could be explained when the studies were considered within their contexts.

### Outcome of translation

Despite variations in healthcare settings and countries, the studies all contributed to an understanding of how healthcare can address or contribute to the harms of sexual violence. Conceptually, most reports framed re-traumatising experiences of healthcare as an individual issue. However, some also explored systemic and structural sources of harm and re-victimisation; these are described in the *objectified and* *dehumanised* sub-theme.

### Outcome of the synthesis process

We generated three themes: (1) *name the violence*, with sub-themes*: shamed and silenced, searching for physical injuries,* and *acknowledgement is a journey*; (2) *make sexual violence visible*, with sub-themes: *unspoken and unheard, a harmful system,* and *ask, validate, respond*; (3) *bear witness*, with sub-themes: *a second attack, objectified and dehumanised*, and *be alongside me*. We also developed a line of argument synthesis, illustrating that demonstrating trustworthiness was central to promoting healing within healthcare settings. Each theme outlines an aspect of how trustworthiness can be demonstrated in healthcare settings. Table 2 provides a summary and CERQual assessment outcome for each theme (Appendix D).

**Table 2:** Summary of each theme and the CERQual assessment outcome

**Line of argument: **Demonstrating trustworthiness was central to trauma-informed healthcare. Trust was earned by creating care environments and interactions that countered the harms of sexual violence: supporting survivors to name and make sense of the violence to counter blame and shame; hearing and believing survivors to challenge silencing and invisibility; and affirming dignity, agency and autonomy to address the dehumanising nature of sexual violence. Trust-building could be undermined by system-level factors that de-prioritised relationships and/or disrupted consistency and accountability. Trust was also prevented and eroded by intersecting forms of oppression that shaped access, recognition, and experiences, compounding barriers and intensifying harm for marginalised and disadvantaged survivors.		
Theme	Sub-themes	CERQual Assessment Outcome
**Theme 1: Name the violence.** Sexual violence was often hidden, minimised, or normalised, which meant that many survivors struggled to acknowledge what had happened to them. Survivors needed time, safety, and gentle support to help them reach this understanding. Being able to name the violence was an important step toward healing, as it challenged internalised blame and broke through the silencing imposed on them (*n* = 34; 68% of included studies).	Shamed and silenced (*n* = 28; 56% of included studies).	High confidence
	Searching for physical injuries (*n* = 7; 14% of included studies).	Moderate confidence
	Acknowledgement is a journey (*n* = 21; 42% of included studies).	High confidence
**Theme 2: Make sexual violence visible.** Sexual violence remained invisible in healthcare settings that lacked safety for disclosure, failed to recognise it as a health issue, or imposed frameworks that overlooked survivors’ own understandings and trauma context. Survivors needed space to disclose, validation of their experiences, and responses that acknowledged trauma and recognised sexual violence as a gendered violation of human rights (*n* = 41; 82% of included studies).	Unspoken and unheard (*n* = 25; 50% of included studies).	High confidence
	A harmful system (*n* = 30; 60% of included studies).	High confidence
	Ask, validate, respond (*n* = 24; 48% of included studies).	High confidence
**Theme 3: Bear witness.** When consent was disregarded, distress dismissed, or providers' own need for control took precedence over survivors' autonomy, it could reproduce the violation, dehumanisation, and silencing of sexual violence. Survivors needed to be treated as whole people, with empathy, respect for autonomy, and dignity. Attuned, respectful care promoted healing through rebuilding trust, restoring agency, and affirming survivors’ worth and humanity (*n* = 43; 86% of included studies).	A second attack (*n* = 20; 40% of included studies).	High confidence
	Objectified and dehumanised (*n* = 23; 46% of included studies).	High confidence
	Be alongside me (*n* = 39; 78% of included studies).	High confidence

#### Theme 1: Name the violence

Sexual violence was hidden, stigmatised, and normalised which made it difficult for survivors to acknowledge their experiences. Acknowledgement was described as a gradual process; survivors needed time, patience, and gentle encouragement to help them come to understand and name certain sexual experiences as violence. This recognition was often a necessary step before they felt able to articulate, make sense of, or seek support for what had happened.

**Shamed and silenced.** Survivors reported feeling shamed and silenced when presenting to healthcare - feelings often rooted in gendered stereotypes that blame women for sexual violence [66, 68, 70, 72, 74, 76, 79, 85, 97, 98, 102, 105, 108]. Survivors’ experiences of being shamed and silenced were shaped not only by gendered stereotypes but also by intersecting histories of racism and colonialism, as well as by the dynamics of experiencing sexual violence within intimate relationships. African American and Black women were uniquely shamed and de-legitimised as sexual violence victims by pervasive racist stereotypes that hyper-sexualised them, rooted in histories of slavery and colonialism [97, 98]. Experiencing sexual violence within a relationship eroded survivors’ self-worth over time; it was intertwined with ongoing coercive control, psychological abuse, and a partner’s sense of entitlement to sex, while also producing shame and guilt linked to parental responsibility and the relationship context - such as feeling responsible for children’s wellbeing or judged for staying in an abusive relationship or not leaving sooner [67, 79, 96, 105].

Survivors often described shame as embodied, such as feeling dirty, untouchable, incomplete, or broken [67, 68, 74, 76, 87, 96, 108], and trauma manifesting as physical symptoms, such as pain, low energy, and repeated gynaecological problems [105, 107]. For some survivors, becoming pregnant or having to take medication for HIV acquired as a result of rape served as an embodied reminder of the violence they had endured as well as the shame associated with it [66, 67, 89, 91].

Women often blamed themselves [67, 70, 77, 83, 98, 105, 108] and expected to be blamed or invalidated by providers [67, 68, 73, 79, 83, 95, 98, 101, 108] which prevented disclosure [67, 83, 98, 101, 108]. Across the studies, many survivors experienced responses to disclosure that re-enforced shame and discouraged them from seeking further support, including responses that minimised the violence, accused them of lying, blamed them, or pitied them [60, 65–68, 74, 81, 84, 85, 91, 92, 96, 98, 105].

**Searching for physical injuries.** Providers, especially those assessing injuries following sexual violence, held enormous power to validate or invalidate women’s experiences. This was a small but important sub-theme that was largely (but not exclusively) informed by studies on Sexual Assault Nurse Examiners (SANE) and/or Medical Forensic Examinations (MFEs) [70, 71, 73]. To acknowledge their experiences as sexualised *violence*, survivors needed both to believe—and to be reassured by providers—that the responsibility for the violence lay with the perpetrator(s), and that they did not deserve and could not have prevented what had happened to them [70, 71, 73, 76, 81]. However, stereotypes of ‘real rape’ being physically violent [77, 81] shaped assumptions among both women and providers that a lack of physical violence or injuries must mean survivors were lying, misremembering, or overreacting – or that the violence was not serious [65, 71, 73, 81]. For instance, a survivor in one study likened the MFE to a lie detection test [71]. Survivors felt validated when healthcare providers identified physical injuries and attributed them to sexual violence [71, 73, 76], but even in the absence of injuries could feel validated if providers actively communicated belief and affirmed the seriousness of the violence [70]. When providers did not identify or attribute injuries to sexual violence and did not offer reassurance or validation, or actively discredited survivors due to the absence of physical injuries, many survivors felt their accounts were doubted, leaving them invalidated, questioning their memories, and blaming themselves [65, 71, 73, 76, 77].

**Acknowledgement is a journey.** Acknowledging sexual violence was important for healing, but this took time - from months to years to decades [74, 76, 83, 96, 99] and was not a linear process [74, 76, 83, 96]. Those experiencing sexual violence from a partner needed to acknowledge the abuse in order to feel ready to leave the relationship, yet were prevented from doing so by its normalisation and the disorienting confusion of being abused by someone they loved and were committed to [79, 96, 103]. At the point of accessing healthcare, women usually did not have the language to name their experiences as violence [76, 83, 96]; some noted that unwanted sexual experience(s) had felt wrong or weird, while others coped by blocking out or minimising their experiences [67, 74, 76, 83, 96].

When disclosing or seeking support, survivors needed providers to recognise non-verbal cues, respond to subtle hints with curiosity and direct questions, and listen carefully to expressions of fear or discomfort [76, 81, 99, 105]. After disclosure, survivors needed providers to support them to acknowledge that they had experienced violence by believing them and validating their distress [70–74, 76, 78, 79, 81, 84, 92, 96, 100, 107], gently naming sexual violence [70, 78, 81, 107], and affirming the seriousness of what happened [70, 71, 73, 74, 76, 81, 84, 92, 96, 100, 108]—without pressuring them into taking actions they are not ready for, such as reporting or leaving an abusive relationship [79, 83, 85, 96, 100]. Supporting women to acknowledge these experiences as *violence* – and therefore not their fault – refuted victim-blaming messages that were exploited by perpetrators, re-enforced by society, and internalised by survivors [76, 78, 79].

#### Theme 2: Make sexual violence visible

Sexual violence was rendered invisible in healthcare settings when disclosures were prevented or when sexual violence was not considered a health-related issue. Providers and services could make sexual violence visible by asking, validating, and responding, underpinned by an understanding of sexual violence as a gendered violation of human rights.

**Unspoken and unheard.** As explored in theme 1, survivors experienced multiple layers of shaming and silencing, preventing acknowledgement and disclosure. Healthcare settings often did not provide the safety and reassurance that survivors needed to feel safe enough to disclose in this context. Survivors were rarely asked about sexual violence or its impacts on them [105], even when a trauma or sexual violence history was known to providers [62, 86]. Structural features of healthcare delivery - such as frequent interruptions and lack of privacy [62, 86, 95], rushed appointments [62, 81, 105], and lack of continuity of care [64, 86, 106] - combined with relational cues like provider disinterest or reluctance [60, 68, 84, 100, 103, 107], prevented survivors from feeling safe enough to introduce the topic themselves.

Some groups experienced additional, and often intersecting, barriers to speaking about sexual violence in health settings. Latina women experiencing partner abuse and raising children feared consequences from multiple systems, including social services and immigration, and therefore needed clarity about what would happen if they spoke to a healthcare provider before they could feel safe enough to do so [105]. Black, African American and Latina women reported being prevented from disclosing by intersecting and systemic harms of racism and sexism, which shaped the discriminatory responses they anticipated from health services [97, 98, 105]. For instance, one Black woman questioned why she would seek medical help for “something as crazy-making as rape” given her lived and historical experiences of a racist and sexist medical system [97] (p. 1275). Women experiencing sexual violence from a partner reported being silenced both by the perpetrator’s physical presence at appointments and by the threat of escalated violence as a form of punishment if they disclosed [67, 96, 105]. Survivors accessing mental health services feared being given additional psychiatric diagnoses or prescribed medication if they disclosed [83].

Even when survivors told healthcare providers about their experiences of sexual violence, they were often not heard. Experiences of having disclosures dismissed, ignored or invalidated were commonly reported, leading to isolation and disengagement [60, 68, 79, 81, 82, 92, 98, 100, 105, 107]. Black and African American women described responses that de-legitimised them as victims, shaped by intersecting forms of silencing rooted in histories of slavery and colonialism, and rendered their trauma invisible due to a failure of services to recognise sexual violence and its impacts as shaped by intersecting oppressions and historical trauma [68, 85, 97, 98]. Survivors of partner-perpetrated sexual violence described a double invisibility due to gendered stereotypes that minimised sexual violence from male partners as well as those that dismissed women experiencing abuse from an intimate partner as dramatic or irrational [79, 96, 105]. Survivors also described feeling unheard when their insights and knowledge were deprioritised in favour of those of providers or the system - for example, when their difficulties were interpreted through system-defined categories, reinforcing an expert–patient hierarchy that invalidated their own knowledge and understanding of their experiences [62, 81, 86, 87, 90–92, 105].

**A harmful system.** The support, validation and care that survivors needed was often not available to them. Survivors needed stability and consistency, but many experienced fragmented and inconsistent care and long waiting times which felt disorientating and prevented them from accessing the support they needed [59, 62–64, 81, 82, 92, 93, 103]. Post-disclosure, survivors reported being shuffled from service to service or needing to repeatedly disclose to multiple providers in an (often futile) attempt to access appropriate support [65, 66, 81, 82, 91–93, 105]. As trauma could impact memory and concentration, survivors needed clear, repeated, and consistent communication to help them make informed decisions [59, 62, 65, 66, 70, 72, 82, 88, 95]. Instead, many survivors reported experiencing chaotic healthcare settings and poor communication that added to their distress [59, 60, 86, 87, 92].

Black and African American survivors reported inequalities in terms of how safe they felt to access healthcare and how they were treated when they did [97]. Black and African American women described feeling unsafe and unwelcome in White-dominated healthcare and support settings [97, 98] and excluded from specialist sexual violence services [68]. Pregnant refugee women facing multiple disadvantages were left to navigate an unfamiliar maternity system alone and further harmed by hostile immigration policies that imposed charges for maternity care [86]. In contexts where healthcare access depended on financial resources, survivors lacking such means were unable to obtain safe abortions for rape-related pregnancies or receive therapy to address the trauma and psychological distress of sexual violence [63, 66, 85, 89].

Particularly relevant to mental health settings was a poor understanding of the complexities of sexual violence and its impacts among providers and services that led to a tendency to pathologise sexual violence as well as trauma responses and symptoms [76, 79, 81–83, 85, 92, 93, 96, 99], such as survivors being framed as dysfunctional [79] or mentally ill [96]. The erasure of sexual violence and its psychological consequences denied survivors access to the care they needed, leaving their trauma unaddressed by services they expected would be equipped to respond, with some medicated but otherwise unsupported [79, 81, 92, 93, 96]. The dominance of the biomedical model in mental health settings left little space for mental health difficulties to be understood in the context of sexual violence or for survivors’ own perspectives on their difficulties and needs to be heard, ultimately hindering survivors from processing their experiences, making sense of them, and moving forward in healing [81, 92].

**Ask, validate, respond.** For survivors to feel safe enough to disclose, it was essential that providers actively signaled their openness to disclosures and demonstrated knowledge of how to respond appropriately. When survivors were asked about sexual violence directly yet sensitively, and when it was made visible in healthcare settings - for example, through posters or flyers - this conveyed care and, for some, provided the support needed to begin acknowledging that they had experienced something violent and unjust [81, 96, 99, 105, 107, 108]. Overall, survivors reported wanting to be asked about sexual violence even if they were not ready to disclose [62, 81, 96, 99, 100, 105, 107, 108]. To feel safe enough to disclose, survivors needed to feel able to choose when, how, and to whom they told and needed to trust that they had control over the consequences of disclosure [62, 74, 79, 89, 105, 107].

Post-disclosure, in addition to what was discussed in theme 1, women needed providers to show empathy [62, 70, 72, 76, 77, 79, 81, 84, 85, 91–93, 96, 100, 105, 107], demonstrate their knowledge of trauma and options for support [62, 70, 73, 77, 81, 84, 85, 92, 93, 96]; normalise survivors’ distress and ways of coping [76, 79, 81, 84, 85]; and view sexual violence as a violation of human rights [62, 69, 77, 81, 91]. African American women needed providers to acknowledge and understand how trauma from racism intersected with trauma from sexual violence [98]. Survivors experiencing intimate partner violence needed providers to understand the dangers of leaving an abusive relationship as well as the unique concerns of women who experience sexual violence within a committed relationship and from men they love [79, 93, 105]. Survivors needed providers to respond in accordance with their level of readiness, which required providers to set aside their own assumptions and listen with the intent to understand rather than to respond [62, 79, 81, 83–85, 89, 105, 107]. Women needed providers to be attuned to their holistic needs, to trust in their capacity to heal, to support their choices, and to resist attempts to ‘fix’ what could not be fixed [62, 69, 70, 72, 73, 79, 83, 84, 91–93, 99, 100, 105, 107].

#### Theme 3: Bear witness

Experiences where survivors’ rights to autonomy, dignity, and respect were not upheld in healthcare could replicate the violation, dehumanisation, and silencing inherent in sexual violence. Survivors needed providers to recognise them as whole persons, demonstrate empathy, support their decision-making, and prioritise relationship-building

**A second attack**. Aspects of care or the actions of providers could feel like, or re-create, the violation and loss of autonomy inherent in sexual violence. On an individual level, survivors reported re-living memories of sexual violence (re-traumatisation) during procedures or examinations that crossed body boundaries or involved sensitive body parts, such as gynaecological examinations, and when experiencing a loss of control over their body, such as during birth, surgery, dental treatment, or restraint [75, 76, 80, 82, 87, 90, 91, 99, 100, 104, 107].

Providers’ failure to seek informed consent was key to explaining how healthcare-related events, procedures, examinations, or processes led to re-traumatisation. When survivors were subjected to touch, procedures, or examinations *without* full, informed consent, they were left feeling violated, ashamed, and subjected to a second attack [68, 69, 71, 73, 82, 87, 90, 96, 99, 100]. However, when survivors felt able to set the pace, felt respected, could say no, and felt in control, the same procedures did not leave them feeling powerless, violated, or re-traumatised in the same way, even if those procedures were uncomfortable, distressing, or invasive [71, 73, 75, 87].

On a systemic level, power imbalances shaped by medical authority led to situations where survivors felt pressure to comply with care they did not want or understand, resulting in a sense of them having procedures and examinations done to them instead of them being active participants in their care [62, 65, 68, 71, 73, 75, 81, 87, 90–92, 99, 100]. Some survivors feared, or were threatened with, being denied care if they declined procedures, and, particularly in maternity care and mental health settings, reported being threatened with, or subjected to, coercion if they did not comply [62, 80, 82, 87, 90, 91]. When faced with these power imbalances, survivors described needing to take the path of least resistance - or to surrender - to protect themselves from further harm or to access the care they needed [62, 87, 90].

**Objectified and dehumanised.** Survivors reported that healthcare settings and interactions with providers re-created the power dynamics of sexual violence, including feeling objectified (reduced to an object, body, body parts, case or diagnosis) and dehumanised (having humanity and/or dignity denied). Survivors described being made to feel like a “slab of meat” [69] (p. 7), “rubbish off the streets’” [75] (p. 690), a “vessel” [82] (p. 5) or “slaughtered animal” [87] (p. 186). This sense of objectification and dehumanisation was reinforced when survivors felt healthcare providers failed to acknowledge them as people: speaking at them rather than to them, issuing instructions with indifference to the impact on their wellbeing, and carrying out tasks with emotional detachment [59, 65, 68, 69, 71, 73, 76, 81, 82, 87, 92, 93, 99, 100, 105]. In mental health settings, when distress or trauma resulting from sexual violence was framed as a pathology located within a survivor, this simultaneously reinforced societal messages that survivors were defective while denying them the empathy and support they needed [62, 79, 81, 92, 96]

Experiences of dehumanisation were gendered and racialised. Women described gendered experiences of humiliation and degradation when their bodies were exposed, examined, or restrained by providers [75, 80–82, 87]. For instance, women survivors with intellectual disabilities who lived on locked wards described male providers’ approach to restraint as more humiliating, more authoritarian and less predictable compared to female providers [80]. A tendency to frame a survivor’s distress as an individual pathology was described by some as gendered, reflecting stereotypes of women as irrational or overly emotional [96, 105].

For Black and African American survivors, experiences of dehumanisation in healthcare settings were intensified by the intersecting oppressions of racism and sexism [97, 98]. This included direct experiences of racialised and gendered humiliation and disrespect, such as White healthcare providers laughing at a Black woman who sought care after rape [97]. The invalidation, neglect and exclusion described by Black, African American, and migrant survivors, as discussed in themes 1 and 2, not only denied them the support they needed and deserved, but also sent a message that they were not considered worthy of care, attention, or empathy [86, 97, 98]. Authors contextualised these experiences of dehumanisation in healthcare within the wider historical dehumanisation of Black and African American women rooted in slavery and its racist legacies, and reinforced by systemic racism in healthcare [97, 98].

**Be alongside me.** To counter the dehumanisation and silencing of sexual violence, survivors needed to feel treated as a whole person rather than a body, case, number, patient, or diagnosis [64, 69–73, 75–77, 79–81, 83, 84, 87, 89, 91, 92, 95–97, 99, 105, 107]. Survivors felt respected when healthcare providers acknowledged the potential for procedures to cause distress by explaining what they were doing and why [60, 61, 68–71, 73, 75, 80, 86, 88, 91, 92, 99, 100]. Survivors emphasised the importance of follow-up and having a designated healthcare provider, which provided a sense of reassurance and accountability [62, 69, 72, 74, 81, 86, 92, 99].

Some survivors noted that there was one individual or service that stood out from a sea of professionals and systems that had dismissed or misunderstood them [69, 79, 81, 86, 88, 92, 98, 99, 106, 108]. For instance, one survivor described a service as an oasis in the desert [92]. While women needed patience, time to process, and space to connect with their own needs [62, 74], the presence of a supportive companion could remind them of life before the assault and help to buffer trauma responses such as flashbacks and numbing [70–72, 74, 75, 80, 99]. Feeling cared for did not require verbal communication and was often described as feeling a comforting human presence, such as experiencing a caring touch or simply having company [59–61, 65, 70–75, 77, 80, 86, 92, 96, 99]. Generally, women survivors described feeling emotionally and physically safer with women compared to men [60, 61, 65, 72, 73, 79, 81, 87, 90, 103], with one study reporting that this preference remained even among women who had experienced sexual violence from a woman [108]. However, this was not a universal experience, as Black women could feel unsafe with White women [97].

#### Line of argument: demonstrating trustworthiness

Each theme was connected by the idea that trauma-informed healthcare needs to be underpinned by a process of reciprocal trust: to promote healing, healthcare providers, services and systems need to earn trust by demonstrating trustworthiness. Each theme illustrates a way in which trustworthiness could be demonstrated in healthcare settings, each counteracting key harms of sexual violence. When providers supported survivors to name their experiences as violence, provided space and time for them to reach this understanding, and gently challenged stereotypes that blamed them, survivors reported feeling able to (re)gain trust in their own memories, feelings, and bodies. When providers appeared curious, knowledgeable, and receptive to survivors’ perspectives, understandings and holistic needs, they signalled care to survivors and that they could be trusted with, and respond appropriately to, disclosures. When providers treated survivors with respect and dignity, affirming survivors’ agency and validating their needs and experiences, they counteracted the deeply dehumanising nature of sexual violence, restoring choice and control and sense of safety with other people.

Across all themes, trustworthiness was demonstrated when providers were attuned, consistent, and responsive, and oriented toward listening and understanding rather than explaining or fixing. However trustworthiness was not only demonstrated relationally through interactions between providers and survivors. It was also shaped by system-level factors, such as providers having the time, encouragement, resources, confidence and knowledge to respond in ways that actively counteracted the dynamics and impacts of sexual violence. Trust was undermined not only by structural conditions - such as fragmented care, lack of continuity, rushed or dismissive interactions, de-prioritising or disregarding consent, and approaches that imposed explanations over listening to survivors - but also by intersecting forms of oppression that shaped access and legitimacy. These overlapping dynamics compounded silencing, violation, neglect, and harm for marginalised and disadvantaged survivors.

## Discussion

This systematic review synthesised qualitative research exploring healthcare experiences and expectations among women who had experienced sexual violence in adulthood. Across the studies, survivors described how healthcare settings could either mirror, or promote healing from, the harms of sexual violence. Together, our findings present a survivor-informed model that can inform trauma-informed approaches in healthcare settings, which places the need to demonstrate trustworthiness at its heart. Our themes and line of synthesis illustrate that trust is earned through counteracting key harms of sexual violence: helping survivors to name and make sense of what happened, which counters silencing and shifts shame and blame (Theme 1); making sexual violence visible, which creates safer environments in which to disclose and access support (Theme 2); bearing witness and prioritising dignity, respect and choice, which counters the loss of agency and dehumanisation associated with sexual violence (Theme 3). Importantly, our synthesis suggests that trust is not only demonstrated through the actions of individual providers, but also through system-level conditions, including power dynamics and broader social inequities that influenced care access, expectations and experiences.

### Challenging the silencing of sexual violence is key to trauma-informed approaches in healthcare

Our review highlights that a central role—and responsibility—of healthcare systems in supporting healing is to challenge the cultural silencing of sexual violence [28, 109]. Sexual violence reduces those subjected to it to “silence, to the status of an object … an instrument of another’s agency” [110] (p. 55). This silencing continues in the aftermath, with taboos around sex [7, 111] and pervasive sexual objectification of women [21] contributing to a sense of sexual violence being a particularly unspeakable type of violence [109]. Fostering agency, voice, and connection is therefore central to healing, often involving a process of survivors internally acknowledging that what happened was violent and not their fault, alongside receiving external validation by others who hear, believe, and affirm their narrative [109, 110, 112, 113]. In our review, healthcare providers and services were framed as holding significant power to challenge the silencing of sexual violence, and, through this, support survivors in beginning to heal. Particularly for those in the early stages of acknowledgement, validation and affirmation in healthcare settings offered a crucial first step before they were ready to seek specialist support. This is important because healthcare providers are often the first or preferred professional point of disclosure following sexual violence [114].

Although healthcare providers and services had the power to challenge silencing, it was more often perpetuated than disrupted in healthcare settings. In the majority of studies, survivors reported feeling repeatedly dismissed and unheard, or described inconsistent and unpredictable responses across multiple providers and services. Although some survivors were made to feel heard and seen by individual providers, these encounters were the exception, with the healthcare system as a whole more often being experienced as invalidating in ways that mirrored the wider societal silencing of sexual violence [109]. While some survivors attempted to disclose despite these barriers, their efforts were frequently overlooked or invalidated, missing an important opportunity to provide support as well as re-traumatising survivors by reinforcing the silencing they encountered elsewhere [11].

Healthcare providers cannot dismantle the cultural silencing of sexual violence alone. They need system-level support to consistently and effectively respond to violence against women [115], including comprehensive and regular training on trauma and violence; violence-specific protocols and policies; and pathways to specialist trauma-focused support [12]. Our findings highlighted systemic conditions – such as rushed interactions and lack of confidential spaces – that are likely to prevent even motivated and knowledgeable providers from sensitively asking about or responding to disclosures of sexual violence. When systems fail to support—or actively undermine—trauma-informed principles, providers may experience despair, moral injury, and burnout which can in turn reinforce re-traumatising practices such as emotional distancing or reliance on power and control [11, 28]. It is therefore critical to apply trauma-informed principles at the system level – as is their intention [27] – to foster environments that consistently provide validation, enable disclosure, and offer clear pathways to specialist support [28].

### Trauma-informed approaches in healthcare must create safety for disclosure without requiring it

Safety and transparency are key principles of trauma-informed approaches [116], and our findings show how central they were to disclosure. In our findings, survivors were more likely to feel safe enough disclose sexual violence when they felt confident that providers would respond with belief, validation, and understanding, and that they were in control over what happened next. Survivors needed reassurance that healthcare was a legitimate place to seek support for sexual violence, along with clear signals of receptiveness and interest from providers. While similar recommendations have been made in relation to intimate partner violence (IPV) [117], survivors often report that sexual violence feels especially difficult to disclose [7]. This is particularly true when their experiences do not fit stereotypical ideas of ‘real rape’ being physically violent and perpetrated by strangers [16], which is the reality for the majority [2, 5], with intimate partner sexual violence remaining especially invisible [7, 31, 111, 118]. Trauma-informed approaches in healthcare must therefore address both the broader silencing of sexual violence and the disproportionate invisibility of certain forms.

One way in which safety to disclose could be built was through sensitive enquiry. This could signal to survivors that their experiences were important and of interest to providers, even if it did not result in a disclosure at that time. Others have highlighted that routine enquiry of IPV, when done with care and empathy, has additional benefits beyond identification, such as helping survivors feel connected and seen [119]. Viewing sensitive enquiry as a way to convey care and understanding, irrespective of disclosure, may be important to trauma-informed approaches in healthcare settings, aligning with their broader aim of creating conditions that enable disclosure without requiring it [28]. While survivors generally report wanting to be asked about sexual as well as other forms of violence [120–122], providers often report discomfort, needing consistent system-level support and ongoing training to feel ready to ask and respond [115, 123, 124]. Routine enquiry appears to be more commonly implemented for IPV than for sexual violence [125], but there is also evidence suggesting that sexual violence within relationships may remain hidden if survivors are only asked about IPV more generally [31]. The gap between what survivors need to safely disclose sexual violence and what health systems currently provide highlights the urgent need to better support providers, so they can create the conditions that enable disclosure—a core element of trauma-informed approaches [12, 28].

Another key way in which safety to disclose could be built was through wider messaging at the service or organisation level, such as through posters or flyers. This suggests that universal education may be an important part of a trauma-informed approach in healthcare settings, whereby everyone in a population that experiences disproportionate rates of sexual violence and/ or are seen in settings that are likely to see a high number of survivors, is provided with information to identify it and seek support [117]. Such an approach reduces reliance on both disclosure and enquiry while still giving survivors the information, knowledge, and tools needed to seek support [117]. Our findings suggest also that this may signal care and receptiveness, which could lay the foundations for potential later disclosure. However, further research should be conducted, in partnership with survivors, to explore the acceptability and feasibility of such an approach, as well as how this may be implemented in different healthcare settings. In line with a trauma-informed approach [28], it is still critical that providers have the appropriate training, knowledge, and system-level support to enable them to enquire with sensitivity and to respond to disclosures with confidence and empathy when needed.

### Trauma-informed approaches in healthcare should frame re-traumatisation as a systemic problem

In our review, some survivors described being subjected to, or threatened with, explicit violence or coercion in healthcare settings, while others reported more subtle forms of power and control, such as repeated dismissal or the de-prioritisation of informed consent. These findings align with broader calls to make visible ways in which power and control can be enacted within healthcare, and how these processes are shaped by gender, race, and the exercise of medical authority [13, 23, 126]. Yet, while there were exceptions (e.g. [80]) authors tended to interpret survivors’ accounts through an individualised PTSD- and neurobiology-informed lens [127], framing their distress as a reactivation of past trauma, which risked obscuring the present harm and violence described in survivors’ accounts.

Focusing narrowly on the neurobiology of trauma can overlook the broader meaning and impact of re-traumatisation, including how it is actively enabled and perpetuated by systemic and societal conditions [127]. For instance, our findings emphasise that biomedical dominance and the exercise of medical authority are important mechanisms that can simultaneously take power from survivors and normalise this as part of help, treatment, or care [11]. Relatedly, feeling reduced to a body, diagnosis, or object lay at the heart of re-traumatising experiences, mirroring the ways in which women had been objectified and dehumanised through sexual violence [21]. When reduced in these ways, survivors reported feeling stripped of their identity, humanity, and autonomy, echoing how sexual violence itself attacks survivors’ sense of self and “reduces the victim to flesh” [110] (p. 55), and intersecting with other forms of dehumanisation such as racism [98]. These are subtle forms of harm and violence that can be difficult to name as such [126], particularly in healthcare settings which are often presumed to be benevolent and free from harm [23].

These findings highlight an urgent need for trauma-informed approaches to recognise not only how the neurobiological consequences of trauma can surface in healthcare, but also the ways that broader dynamics of power, control and silencing tied to sexual violence [21, 127] - including subtle or less visible forms [6, 31] - may be reproduced – as well as prevented. As an organisational-level intervention and culture change process, trauma-informed approaches are, in theory, designed to address systemic causes of re-traumatisation, such as practices rooted in power, control, and inequity [27–29, 128, 129]. In practice, however, this system focus is often missed, with implementation tending to remain at the level of patient–provider interactions [29]. Moreover, limited attention is often paid to principles intended to balance power and promote agency and dignity, such as collaboration, choice, and strengths-based approaches [130]. Our review showed that limitations in the implementation of trauma-informed approaches were mirrored in how trauma and re-traumatisation in healthcare were conceptualised in research; namely, that harms are often conceptualised individually in practice even when theory indicates the need to look wider than this. Making visible the more subtle ways in which harm from sexual violence is reproduced in healthcare, often at a system level, requires research, policy and practice efforts to move beyond narrow framings of sexual violence as ‘sex minus consent’ [131] and to recognise it as a relational harm - often involving betrayal of trust and disruption of social connection [109] - that is rooted in power [21, 127].

### Trauma-informed approaches to healthcare must embed an equity lens

In our review, experiences of re-traumatisation, harm and healing in healthcare were not uniform, but shaped by broader social and systemic inequities. Black and African American survivors described how intersecting forms of discrimination, particularly at the intersections of race and gender, de-legitimised them as victims of sexual violence, thus preventing access to belief, support, and acknowledgment [98]. They reported being subjected to harmful, racist stereotypes that hypersexualised them and undermined the seriousness of sexual violence perpetrated against them [97, 98, 132]. Migrant survivors also feared disclosure would trigger immigration-related consequences and were left to navigate unfamiliar health systems without support, creating unique layers of silencing [86, 105]. These findings underscore the importance of trauma-informed approaches acknowledging how intersecting systems of oppression and disadvantage as well as historical trauma shape experiences of both sexual violence and re-traumatisation in healthcare [97, 98, 133]. While trauma-informed approaches emphasise a broad definition of trauma, including collective and historical forms [28], this structural dimension is often de-prioritised or overlooked in practice [130, 134].

Our findings suggest that for trauma-informed approaches to effectively prevent harm and promote healing for all survivors, they must adopt an equity lens and actively confront assumptions, norms, and stereotypes—including, but not limited to, those identified in our review—that legitimise some survivors while silencing or rendering others invisible [98]. The perspectives of many disadvantaged, marginalised and minoritised groups were absent from our synthesis, highlighting the urgent need to build an evidence base on how intersecting identities shape experiences, access, and needs in relation to healthcare and sexual violence. Without reflection on these issues, trauma-informed approaches—and the research studies underpinning them—risk universalising experiences of sexual violence and its impacts, further marginalising survivors whose experiences fall outside this narrow frame. Applying an intersectional lens [135] alongside embedding principles of anti-racism, equity, and justice into the design and implementation of trauma-informed approaches is therefore vital to ensuring that disadvantaged and marginalised survivors are appropriately supported to speak about and seek support for sexual violence—and are truly heard when they do.

### Demonstrating trustworthiness is foundational to meeting healthcare’s potential to promote healing

Our line of argument emphasises that *demonstrating* trustworthiness is fundamental to trauma-informed approaches in healthcare that respond effectively to sexual violence against women, and is central to both preventing further harm and promoting healing. Although survivors are often portrayed as lacking the ability to trust [136], our synthesis highlights that the responsibility lies with healthcare systems to demonstrate trustworthiness [136]—including ensuring staff are supported to do the same—so that survivors can feel safe enough to place their trust in them. Trustworthiness is a key principle of trauma-informed approaches [116], as well as being recognised as an essential component of a well-functioning health system [137] that is shaped by the availability of good-quality care [138]. Generally, trust is built on expectations that healthcare providers and systems have appropriate knowledge and skills, that they will act in the patient’s best interest, and that they possess qualities of “beneficence, fairness, and integrity” [139] (p. 92).

In our review, survivors rarely described feeling that healthcare providers, services, or systems were sufficiently motivated, knowledgeable or skilled to respond appropriately to sexual violence – both in terms of disclosures and impacts – and often described injustice and inequity in healthcare settings. Survivors also described having different views of their best interests than those held by providers [11, 140]. When their perspectives and knowledge were dismissed—particularly when provider authority undermined their agency and autonomy—these interactions could be re-traumatising by mirroring earlier experiences of silencing, coercion, or control [11]. Overall, healthcare settings, responses and practices that blamed, dismissed, and took power away from survivors undermined ideas that healthcare providers, services, and systems were knowledgeable, ethical, trustworthy, and had integrity, eroding trust. These findings echo broader concerns about the capacity of health systems to respond effectively to violence against women [11, 12, 19], and underscore the need to embed trauma-informed approaches so that healthcare’s potential to promote healing, as highlighted in our review, can be fully realised.

When trust is not demonstrated, or is actively compromised, survivors may be left to bear the impacts of violence alone, with missed opportunities for secondary prevention through timely, supportive responses. Inadequate or harmful responses may also exacerbate trauma and health complications over time, undermining tertiary prevention efforts aimed at supporting long-term wellbeing. The consequences also extend beyond individual survivor–provider interactions. Our findings suggest that, at a societal level, failure to establish trauma-informed, trustworthy healthcare services risks further eroding trust in health systems, especially among those already disadvantaged and marginalised, reinforcing cycles of exclusion, inequality, and systemic harm.

#### Strengths, limitations, reflexivity

We consider our lived experience standpoint a key strength of this review. It enabled us to centre survivors’ perspectives throughout the analysis [32, 33]—a particularly important contribution given that, in the included studies, survivors were rarely involved in roles beyond being participants (only 4% of included studies). This standpoint also informed our assessment of research quality, prompting us to include additional items in the CASP checklist [52] that reflected survivor priorities, such as attending to issues of power or meaningful involvement of people with lived experience [32]. It also shaped our decision to include grey literature and theses, recognising that survivor-generated knowledge often exists outside traditional academic spaces [141].

Our approach was rigorous and included a comprehensive and systematic search of a large number of databases, appraising studies against two established quality assessment tools, and systematically assessing the confidence in the review findings. Following eMERGe reporting guidelines ensured our meta-ethnography was reported transparently and robustly [34].

Our review is limited by a lack of research in in low- and middle-income countries and with survivors from underserved groups, including but not limited to Black and minoritised survivors, LGBTQIA+ survivors, older survivors and survivors facing multiple and intersecting forms of disadvantage and marginalisation. There is a general lack of diversity in health and violence research which risks rendering the experiences and needs of minoritised and marginalised survivors invisible [142]. We sought to address this by exploring how marginalisation and intersecting systems of oppression shaped survivors’ healthcare experiences and expectations, where such experiences were reported. However, this nuance was often absent from the primary studies, and the perspectives of many minoritised and marginalised groups remain underrepresented in our review. Moreover, even when marginalised survivors were included, authors did not always examine the role of oppression or structural injustice in shaping their experiences of care.

#### Recommendations for practice, policy, and research

Table 3 presents recommendations for trauma-informed healthcare responses to sexual violence across research, systems, and policy, grounded in our synthesis. It also includes researcher-focused recommendations informed by our quality appraisal. These address methodological and ethical limitations in the literature—particularly issues rarely reported but vital to survivors, such as meaningful involvement [32]—and gaps where interpretations overlooked system-level or structural harms critical to understanding re-traumatisation.

**Table 3:** Recommendations for healthcare providers, healthcare systems, and researchers

Healthcare providers	Healthcare systems	Researchers
Build in reflective practice to identify assumptions or beliefs that may create barriers to addressing sexual violence, paying particular attention to beliefs and stereotypes that prevent sensitive responses to underserved groups of survivors.	Addressing sexual violence requires a whole-systems approach. Take a clear stance that sexual violence is unacceptable, and that the health system plays a critical role in addressing it.	Ensure research reflects the priorities of survivors and does not re-enforce harmful stereotypes or assumptions.
Ask directly but gently about sexual violence if you have a sense that someone may have experienced it. Trust that a survivor can say no if they wish.	Co-design services and evaluations in genuine partnership with survivors.	Involve survivors meaningfully in all phases of research (from conception to dissemination). Report how and at what stages survivors were involved in shaping and/or carrying out the research.
Consider asking about (sexual) violence more than once where there are opportunities to do so (e.g., primary care, maternity care, mental health).	Educate all staff about sexual violence. Staff should understand it as a violation of human rights, understand how to apply trauma-informed principles to their practice, and appreciate how social, cultural and historical factors (such as racism, poverty, sexism) and their intersectionality shape survivors’ experiences and expectations of both sexual violence and healthcare.	Apply trauma-informed principles to research design, methods, analysis, interpretations, dissemination and ethical considerations and report how this was done.
View consent-seeking as an ongoing process, not a single act.	Develop and deliver staff education with meaningful input from survivors and/or survivor-led organisations.	Build in to the research an acknowledgement that healthcare settings can contribute to harm, and that survivors’ perspectives are essential to understanding how to prevent, address and repair this harm.
If someone discloses sexual violence, name it. Recognise that survivors are unlikely to use explicit terms such as ‘rape’ or ‘sexual violence’ when disclosing. Feeling heard and validated is key, and being heard may be all a survivor needs from you.	Ensure providers have access to clear and up-to-date information about local options for support	Consider the historical, social and structural contexts that shape experiences and expectations of healthcare and of sexual violence.
Be aware of local support services, including grass-roots organisations, so that you can signpost survivors to support (if the survivor wants this).	Equip providers with the knowledge and resources to enquire about sexual violence, identify it (including when perpetrated by a partner), and discuss options for support (if the survivor wants this).	Critically engage with and reflect on whose experiences are included and whose are missing in the research.
Understand that marginalisation and disadvantage shape experiences and impacts of sexual violence as well as experiences and expectations of healthcare.	Consider whether existing policies could create environments that are distressing for or harmful to survivors and ensure they align with gender-sensitive, trauma-informed and anti-oppressive values and principles.	Carry out research focusing on the experiences, expectations and experiences of survivors facing disadvantage, marginalisation or from underserved communities.
Prioritise choices and listen to survivors’ perspectives. Trust that survivors are best placed to understand their own difficulties and their needs.		Identify how healthcare settings can equip survivors with the knowledge and tools to acknowledge their experiences and seek support without requiring a disclosure.

## Conclusions

Although survivors can feel re-traumatised in healthcare settings, there is significant potential to promote healing. Trauma-informed healthcare helps providers to guide survivors in acknowledging and understanding their experiences of sexual violence; respond to disclosures with empathy and sensitivity and offer appropriate options for assistance. It prioritises survivor choices, autonomy, and agency while also challenging the broader systems of oppression and social inequality that cause disproportionate re-traumatisation and harm for already disadvantaged and marginalised survivors. By addressing survivors holistically and with humanity—as whole people and as equals—healthcare can foster genuine healing. However, as long as sexual violence remains invisible, unaddressed, and misunderstood in health systems, survivors will continue to face practices that replicate the dynamics of sexual violence, preventing them from trusting healthcare providers and services. To overcome the potential for re-traumatisation, solutions must target the systemic and structural factors underlying these issues, necessitating changes to the system, not just individuals. Taken together, our findings highlight an urgent need for the system-wide implementation of trauma-informed approaches in healthcare with survivors’ perspectives and priorities at its core. To achieve genuine change, providers, researchers, policymakers, and system leaders must work from the understanding that trust is earned. It takes time to build and commitment and accountability to repair.

## Electronic supplementary material

Below is the link to the electronic supplementary material.

Supplementary Material 1

Supplementary Material 2

Supplementary Material 3

Supplementary Material 4

Supplementary Material 5

## Data Availability

The datasets used and/or analysed during the current study are available from the corresponding author on reasonable request.
