# Supplementary material for: Trauma-informed healthcare from the perspectives of women who have experienced sexual violence in adulthood: a systematic review and meta-ethnography
Source: BMC Health Serv Res. 2025 Nov 27;26:13. doi: 10.1186/s12913-025-13584-x (PMC12763930; doi:10.1186/s12913-025-13584-x)
Supplement: Supplementary file 1 — Supplementary Material 1 [file 12913_2025_13584_MOESM1_ESM.docx]

**Appendix C. eMERGe Reporting Criteria (reproduced from France et al., 2019a).**

| **No.** | **Criteria Headings** | **Reporting Criteria** | **Page number.** |
| --- | --- | --- | --- |
| Phase 1—Selecting meta-ethnography and getting started | | |  |
| *Introduction* | | |  |
| 1 | Rationale and context for the meta-ethnography | Describe the gap in research or knowledge to be filled by the meta-ethnography, and the wider context of the meta-ethnography | 2-3 |
| 2 | Aim(s) of the meta-ethnography | Describe the meta-ethnography aim(s) | 3 |
| 3 | Focus of the meta-ethnography | Describe the meta-ethnography review question(s) (or objectives) | 3 |
| 4 | Rationale for using meta-ethnography | Explain why meta-ethnography was considered the most appropriate qualitative synthesis methodology | 3 |
| Phase 2—Deciding what is relevant | | |  |
| *Methods* | | |  |
| 5 | Search strategy | Describe the rationale for the literature search strategy | 3 |
| 6 | Search processes | Describe how the literature searching was carried out and by whom | 3 |
| 7 | Selecting primary studies | Describe the process of study screening and selection, and who was involved | 4 |
| *Findings* | | |  |
| 8 | Outcome of study selection | Describe the results of study searches and screening | 7-8 |
| Phase 3—Reading included studies | | |  |
| *Methods* | | |  |
| 9 | Reading and data extraction approach | Describe the reading and data extraction method and processes | 4-5 |
| *Findings* | | |  |
| 10 | Presenting characteristics of included studies | Describe characteristics of the included studies | 8 - 9 |
| Phase 4—Determining how studies are related | | |  |
| *Methods* | | |  |
| 11 | Process for determining how studies are related | Describe the methods and processes for determining how the included studies are related: - Which aspects of studies were compared AND - How the studies were compared | 6 |
| *Findings* | | |  |
| 12 | Outcome of relating studies | Describe how studies relate to each other | 8 |
| Phase 5—Translating studies into one another | | |  |
| *Methods* | | |  |
| 13 | Process of translating studies | Describe the methods of translation**:** - Describe steps taken to preserve the context and meaning of the relationships between concepts within and across studies- Describe how the reciprocal and refutational translations were conducted- Describe how potential alternative interpretations or explanations were considered in the translations | 6 |
| *Findings* | | |  |
| 14 | Outcome of translation | Describe the interpretive findings of the translation. | 9 |
| Phase 6—Synthesising translations | | |  |
| *Methods* | | |  |
| 15 | Synthesis process | Describe the methods used to develop overarching concepts (“synthesised translations”). Describe how potential alternative interpretations or explanations were considered in the synthesis | 6-7 |
| *Findings* | | |  |
| 16 | Outcome of synthesis process | Describe the new theory, conceptual framework, model, configuration, or interpretation of data developed from the synthesis | 9 - 18 |
| Phase 7—Expressing the synthesis | | |  |
| *Discussion* | | |  |
| 17 | Summary of findings | Summarize the main interpretive findings of the translation and synthesis and compare them to existing literature | 18 - 23 |
| 18 | Strengths, limitations, and reflexivity | Reflect on and describe the strengths and limitations of the synthesis: - Methodological aspects—for example, describe how the synthesis findings were influenced by the nature of the included studies and how the meta-ethnography was conducted - Reflexivity—for example, the impact of the research team on the synthesis findings | 23 - 24 |
| 19 | Recommendations and conclusions | Describe the implications of the synthesis | 24 |
