# Supplementary material for: Trauma-informed healthcare from the perspectives of women who have experienced sexual violence in adulthood: a systematic review and meta-ethnography
Source: BMC Health Serv Res. 2025 Nov 27;26:13. doi: 10.1186/s12913-025-13584-x (PMC12763930; doi:10.1186/s12913-025-13584-x)
Supplement: Supplementary file 2 — Supplementary Material 2 [file 12913_2025_13584_MOESM2_ESM.docx]

**Appendix B. Ovid Search Strategy**

1 exp rape/ or rape.mp.

2 sexual violence.mp. or exp sexual violence/

3 sexual abuse.mp. or exp sexual abuse/

4 sexual assault.mp. or exp sexual assault/

5 exp interview/ or interview*.mp.

6 experience*.mp.

7 qualitative.mp.

8 exp qualitative research/ or qualitative research.mp.

9 primary healthcare.mp. or exp primary healthcare/

10 health service.mp. or exp health service/

11 exp sexual health/ or sexual health clinic.mp.

12 exp mental health service/ or mental health service*.mp.

13 exp maternal care/ or maternity service*.mp.

14 perinatal care.mp. or exp perinatal care/

15 exp dentist/ or dentist*.mp.

16 community care.mp. or exp community care/

17 1 or 2 or 3 or 4

18 5 or 6 or 7 or 8

19 9 or 10 or 11 or 12 or 13 or 14 or 15 or 16

20 17 and 18 and 19
