# Supplementary material for: Trauma-informed healthcare from the perspectives of women who have experienced sexual violence in adulthood: a systematic review and meta-ethnography
Source: BMC Health Serv Res. 2025 Nov 27;26:13. doi: 10.1186/s12913-025-13584-x (PMC12763930; doi:10.1186/s12913-025-13584-x)
Supplement: Supplementary file 3 — Supplementary Material 3 [file 12913_2025_13584_MOESM3_ESM.docx]

**Appendix C. CASP and additional quality criteria.**
