# Supplementary material for: Trauma-informed healthcare from the perspectives of women who have experienced sexual violence in adulthood: a systematic review and meta-ethnography
Source: BMC Health Serv Res. 2025 Nov 27;26:13. doi: 10.1186/s12913-025-13584-x (PMC12763930; doi:10.1186/s12913-025-13584-x)
Supplement: Supplementary file 4 — Supplementary Material 4 [file 12913_2025_13584_MOESM4_ESM.docx]

Appendix D. CERQual Evidence Profile.

| Theme 1: Name the violence. The hidden and normalised nature of sexual violence made it difficult for survivors to acknowledge their experiences. Survivors needed time and gentle encouragement to name the violence and begin to heal. Doing so supported healing by refuting victim-blaming messages that silenced and blamed survivors (n = 34). |
| --- |

| Sub-themes | Studies contributing to the sub-theme | Methodological limitations | Coherence | Adequacy | Relevance | Assessment of confidence in evidence | Explanation of CERQual  assessment |
| --- | --- | --- | --- | --- | --- | --- | --- |
| Shamed and silenced. Survivors reported feelings of shame and disorientation when reporting to healthcare; feelings which were often rooted in gendered stereotypes that blame women for sexual violence (n = 28). | 59, 64, 65, 66, 67, 69, 71, 72, 73, 75, 78, 80, 82, 83, 84, 86, 88, 90, 91, 94, 95, 96, 97, 100, 101, 104, 106, 107 | Minor concerns. Ten studies with (36%) no or minor concerns (67, 73, 75, 80, 82, 91, 96, 97, 104, 106); sixteen (57%) with minor concerns (59, 64, 65, 66, 69, 71, 72, 78, 83, 86, 88, 90, 94, 95, 100, 107); and two (7%) with moderate concerns (84, 101). | No or very minor concerns. The sub-theme was well supported across the studies. Any variation between studies was minor and could be explained by contextual differences rather than reflecting contradictory evidence. | No or very minor concerns. A large number of studies contributed to the sub-theme, representing various contexts and settings, with several providing nuanced, conceptually-rich insights. | Minor concerns. Eight studies (29%) focused on partially relevant populations, though five still focused exclusively on sexual violence (64, 69, 83, 90, 96) and three on intimate partner violence (94, 95, 104), which remains highly relevant to the adulthood context of this review. Seven studies (25%) did not focus on healthcare settings (67, 75, 82, 94, 96, 100, 101). Two studies (7%; 94, 96) raised relevance concerns on both dimensions (i.e., they addressed neither healthcare settings nor adulthood sexual violence). | High confidence | Most studies (26 or 93%) had no or minor methodological concerns. There were no or only very minor concerns about coherence and adequacy. A large number of studies contributed to this review finding (28 or 56% of all studies included in the review). Most studies (26 or 93%) were either directly relevant to the review question or raised only minor relevance concerns. The sub-theme was well represented across conceptually rich studies and diverse healthcare settings. |
| Searching for physical injuries. Assumptions that ‘real rape’ must involve physical violence led to assumptions that a lack of physical injuries must mean that survivors were lying, misremembering, or overreacting (n = 7). | 64, 69, 70, 72, 75, 76, 80 | Minor concerns. Three (43%) studies with no or minor concerns (75, 76, 80) and four (57%) with minor concerns (64, 69, 70, 72). | No or very minor concerns. The finding was well supported across the studies. Any variation between studies was minor and could be explained by contextual differences rather than reflecting contradictory evidence. | Moderate concerns. A relatively small number of studies contributed to this sub-theme, and were largely focused on the medical forensic examination. However, they did provide nuanced, conceptually-rich insights. | Minor concerns. Two studies (29%) focused on partially relevant populations (64, 69), though both raised only minor concerns as they still focused exclusively on sexual violence survivors. One study (14%) did not focus on healthcare settings (75). None raised relevance concerns on both dimensions (i.e., all focused either on healthcare settings or adulthood sexual violence, or addressed both). | Moderate confidence | All studies (7 or 100%) had no or minor methodological concerns. No or very minor concerns about coherence. Moderate concerns about adequacy. A relatively small number of studies contributed to this review finding (7, or 14% of all studies included in the review). All (7 or 100%) were either directly relevant to the review question or raised only minor relevance concerns. As most studies focused on the medical forensic examination, and a small number contributed to the finding, this sub-theme provides partial evidence, hence being given a rating of moderate confidence. |
| Acknowledgement is a journey. Survivors often did not have the language to name sexual violence. They needed providers to be alert to non-verbal communication, and to respond to subtle hints with curiosity and direct questions (n = 21). | 66, 69, 70, 71, 72, 73, 75, 77, 78, 80, 82, 83, 84, 91, 95, 98, 99, 102, 104, 106, 107 | Minor concerns. Ten (48%) studies with no or minor concerns (73, 75, 77, 80, 82, 91, 98, 99, 104, 106); ten (48%) with minor concerns (66, 69, 70, 71, 72, 78, 83, 95, 102, 107); and one (4%) with moderate concerns (84), | No or very minor concerns. The finding was well supported across the studies. Any variation between studies was minor and could be explained by contextual differences rather than reflecting contradictory evidence. | No or very minor concerns. A large number of studies contributed to the sub-theme, representing various contexts and settings, with several providing nuanced, conceptually-rich insights. | Minor concerns. Six studies (29%) focused on partially relevant populations, though all raised only minor relevance concerns. Specifically, four studies still focused exclusively on sexual violence survivors (69, 83, 98, 99), and two examined intimate partner violence (95, 104), which remains highly relevant to the adulthood context of this review. Three studies (14%) did not focus on healthcare settings (75, 82, 102). None raised relevance concerns on both dimensions (i.e., all focused either on healthcare settings or adulthood sexual violence, or addressed both). | High confidence | Most studies (20 or 95%) raised no or minor methodological concerns. There were no or very minor concerns about coherence or adequacy. A large number of studies contributed to this review finding (21, or 42% of all studies included in the review). All were either directly relevant to the review question or raised only minor relevance concerns. The sub-theme was well represented across conceptually rich studies and diverse healthcare settings. |

| Theme 2: Make sexual violence visible. Sexual violence was rendered invisible in healthcare settings when disclosures were prevented or when sexual violence was not considered a health-related issue. Providers and services could make sexual violence visible by asking, validating, and responding, and understanding sexual violence as a gendered violation of human rights (n = 41 studies). |
| --- |

| Sub-themes | Studies contributing to the sub-theme | Methodological limitations | Coherence | Adequacy | Relevance | Assessment of confidence in evidence | Explanation of CERQual  assessment |
| --- | --- | --- | --- | --- | --- | --- | --- |
| Unspoken and unheard. Healthcare settings often did not provide the safety needed to disclose. Even when survivors overcame substantial barriers to disclosure, they were often not heard or understood (n = 25). | 59, 61, 63, 66, 67, 78, 80, 81, 82, 83, 84, 85, 86, 89, 90, 91, 94, 95, 96, 97, 99, 102, 104, 105, 106 | Minor concerns. Twelve studies (48%) with no or minor concerns (61, 67, 80, 81, 82, 89, 91, 96, 97, 99, 104, 106); eleven (44%) with minor concerns (59, 66, 78, 83, 85, 86, 90, 94, 95, 102, 105); and two (8%) with moderate concerns (63, 84). | No or very minor concerns. The finding was well supported across the studies. Any variation between studies was minor and could be explained by contextual differences rather than reflecting contradictory evidence.. | No or very minor concerns. A large number of studies contributed to the sub-theme, representing various contexts and settings, with several providing nuanced, conceptually-rich insights. | Minor concerns. Twelve studies (48%) focused on partially relevant populations, though most of these raised only minor concerns. Specifically, seven studies still focused exclusively on sexual violence survivors (81, 83, 89, 90, 96, 99, 105), and four examined intimate partner violence (63, 94, 95, 104), which remains highly relevant to the adulthood context of this review. One study included a mixed sample that was not specifically violence-focused (85). Seven studies (67, 82, 89, 94, 96, 102, 105) did not focus on healthcare experiences. Four studies (89, 94, 96, 105) raised relevance concerns on both dimensions (i.e., they addressed neither healthcare settings nor adulthood sexual violence). | High confidence | Most studies (23 or 92%) had no or minor methodological concerns. There were no or very minor concerns about coherence or adequacy. A large number of studies contributed to this review finding (25, or 50% of all studies included in the review). The majority (20 or 80%) were directly relevant to the review question or raised only minor relevance concerns. The sub-theme was well represented across conceptually rich studies and diverse healthcare settings. |
| A harmful system. Survivors described fragmented services and poor communication which deepened distress. Marginalised groups faced exclusion and misunderstanding, and a tendency to pathologise trauma left many survivors unsupported (n = 30). | 58, 59, 61, 62, 63, 64, 65, 67, 69, 71, 75, 78, 80, 81, 82, 84, 85, 86, 87, 88, 90, 91, 92, 94, 95, 96, 97, 98, 102, 104 | Minor concerns. Twelve (40%) with no or minor concerns (61, 62, 67, 75, 80, 81, 82, 91, 96, 97, 98, 104); sixteen (53%) with minor concerns (58, 59, 64, 65, 69, 71, 78, 85, 86, 87, 88, 90, 92, 94, 95, 102); and two (7%) with moderate concerns (63, 84). | No or very minor concerns. The finding was well supported across the studies. Any variation between studies was minor and could be explained by contextual differences rather than reflecting contradictory evidence. | No or very minor concerns. A large number of studies contributed to the sub-theme, representing various contexts and settings, with several providing nuanced, conceptually-rich insights. | Minor concerns. Fourteen studies (47%) focused on partially relevant populations, though most of these raised only minor concerns. Specifically, nine studies still focused exclusively on sexual violence survivors (58, 62, 64. 69, 81, 87, 90, 96, 98), and four examined intimate partner violence (63, 94, 95, 104), which remains highly relevant to the adulthood context of this review. One study had a mixed sample that was not focused on violence (85). Six studies (20%) did not focus on healthcare settings (67, 75, 82, 94, 96, 102). Two studies (7%; 94, 96) raised relevance concerns on both dimensions (i.e., they addressed neither healthcare settings nor adulthood sexual violence). | High confidence | Most studies (28 or 93%) had no or minor methodological concerns. There were no or very minor concerns about coherence or adequacy. A very large number of studies contributed to this review finding (30, or 60% of all studies included in the review). The majority (27 or 90%) were directly relevant to the review question or raised only minor relevance concerns. The sub-theme was well represented across the most conceptually rich studies and diverse healthcare settings. |
| Ask, validate, respond. Survivors described needing providers to demonstrate their receptiveness to disclosures, and to respond with empathy and alignment with survivors’ level of readiness (n = 24). | 61, 68, 69, 71, 72, 73, 75, 76, 78, 80, 82, 83, 84, 88, 90, 91, 92, 95, 97, 98, 99, 104, 106, 107 | Minor concerns. Thirteen (54%) with no or minor concerns (61, 68, 73, 75, 76, 80, 82, 91, 97, 98, 99, 104, 106); ten (42%) with minor concerns (69, 71, 72, 78, 83, 88, 90, 92, 95, 107); and one (4%) with moderate concerns (84). | No or very minor concerns. The finding was well supported across the studies. Any variation between studies was minor and could be explained by contextual differences rather than reflecting contradictory evidence. | No or very minor concerns. A large number of studies contributed to the sub-theme, representing various contexts and settings, with several providing nuanced, conceptually-rich insights. | Minor concerns. Eight studies (33%) focused on partially relevant populations, though all of these raised only minor concerns. Specifically, six studies still focused exclusively on sexual violence survivors (68, 69, 83, 90, 99, 98), and two examined intimate partner violence (94, 95, 104), which remains highly relevant to the adulthood context of this review. Two studies (8%) did not focus on healthcare settings (75, 82). None raised relevance concerns on both dimensions (i.e., all focused either on healthcare settings or adulthood sexual violence, or addressed both). | High confidence | Most studies (23 or 96%) had no or minor methodological concerns. There were no or very minor concerns about coherence or adequacy. A large number of studies contributed to this review finding (24, or 48% of all studies included in the review). All were directly relevant to the review question or raised only minor relevance concerns. The sub-theme was well represented across the most conceptually rich studies and diverse healthcare settings. |

| Theme 3: Bear witness. When survivors' right to autonomy, dignity, and respect was violated or disregarded this could re-create the violation, dehumanisation and silencing of sexual violence. Survivors needed providers to treat them as whole people, show empathy, support them to make choices, and prioritise relationship-building (n = 43 studies). |
| --- |

| Sub-themes | Studies contributing to the sub-theme | Methodological limitations | Coherence | Adequacy | Relevance | Assessment of confidence in evidence | Explanation of CERQual  assessment |
| --- | --- | --- | --- | --- | --- | --- | --- |
| A second attack. Survivors described healthcare experiences that re-created the dynamics of sexual violence, leading to re-traumatisation. These included procedures that crossed body boundaries, removed control, or disregarded informed consent, often exacerbated by power imbalances and medical authority (n = 20). | 61, 64, 67, 68, 70, 72, 74, 75, 79, 80, 81, 86, 89, 90, 91, 95, 98, 99, 103, 106 | Minor concerns. Thirteen (65%) with no or minor concerns (61, 67, 68, 75, 79, 80, 81, 89, 91, 98, 99, 103, 106) and seven (35%) with minor concerns (64, 70, 72, 74, 86, 90, 95). | Minor concerns. Differences in disciplinary framing were noted. Health-focused studies often emphasised individualised, PTSD-focused or biomedical explanations of trauma and re-traumatisation, whereas sociological and feminist studies highlighted relational, systemic and structural dynamics. Our synthesis integrated these perspectives. | No or very minor concerns. A large number of studies contributed to the sub-theme, representing various contexts and settings, with several providing nuanced, conceptually-rich insights. | Minor concerns. Nine studies (45%) focused on partially relevant populations, though most of these raised only minor relevance concerns. Specifically, seven studies focused exclusively on sexual violence survivors (64, 68, 81, 89, 90, 98, 99) and one examined intimate partner violence (95), which remains highly relevant to the adulthood context of this review. One study included a mixed sample that was not violence focused (79). Four studies did not focus on healthcare settings (67, 103, 89, 75). One study (89) raised relevance concerns on both dimensions (i.e., it addressed neither healthcare settings nor adulthood sexual violence). | High confidence | All studies (20 or 100%) had no or minor methodological concerns. There were minor concerns about coherence, which could be explained by differences in disciplinary framing. There no or very minor concerns about adequacy. A large number of studies contributed to this review finding (20, or 40% of all included studies). The majority (18 or 90%) were directly relevant to the review question or raised only minor relevance concerns. The sub-theme was well represented across the most conceptually rich studies and diverse healthcare settings. |
| Objectified and dehumanised. Survivors described healthcare interactions that mirrored the power dynamics of sexual violence, leaving them feeling objectified and dehumanised. This included feeling treated as a case, body, or a diagnosis. Feeling objectified and dehumanised could also feel gendered and racialised (n = 23). | 58, 61, 64, 67, 68, 70, 72, 74, 75, 78, 79, 80, 81, 85, 86, 91, 92, 95, 96, 97, 98, 99, 104 | Minor concerns. Thirteen (57%) with no or minor concerns (61, 67, 68, 75, 79, 80, 81, 91, 96, 97, 98, 99, 104) and ten (43%) with minor concerns (58, 64, 70, 72, 74, 78, 85, 86, 92, 95). | No or very minor concerns. The finding was well supported across the studies. Any variation between studies was minor and could be explained by contextual differences rather than reflecting contradictory evidence. | .Minor concerns. While relatively few studies explicitly addressed power and oppression, incorporating and amplifying these intersecting experiences was critical to ensuring analytical depth, conceptual richness, and alignment with the survivor-centred aims of the review. | Minor concerns. Eleven studies (48%) included partially relevant populations, though most of these raised only minor concerns. Specifically, seven studies still focused exclusively on sexual violence survivors (58, 64, 68, 81, 96, 99, 98), and two examined intimate partner violence (95, 104), which remains highly relevant to the adulthood context of this review. Two studies included mixed samples that were not specifically violence-focused (79, 85). Three studies (13%) did not focus on healthcare settings (67, 75, 96). One study (96) raised relevance concerns on both dimensions (i.e., it addressed neither healthcare settings nor adulthood sexual violence). | High confidence | All studies (100%) had no or minor methodological concerns. There were no or very minor concerns about coherence and minor concerns about adequacy. A large number of studies contributed to this review finding (23, or 46% of all included studies). The majority (20 or 87%) were either directly relevant to the review question or raised only minor relevance concerns. The sub-theme was well represented across the most conceptually rich studies and diverse healthcare settings. |
| Be alongside me. To counteract the dehumanisation of sexual violence, survivors needed to feel treated as a whole person and feel cared about. Some noted that there was one individual or service that treated them with respect and kindness, marking a turning point in their healing journey (n = 39). | 58, 59, 60, 61, 63, 64, 67, 68, 69, 70, 71, 72, 73, 74, 75, 76, 78, 79, 80, 82, 83, 85, 86, 87, 88, 89, 90, 91, 94, 95, 96, 97, 98, 99, 102, 104, 105, 106, 107 | Minor concerns. Seventeen (43%) with no or minor concerns (61, 67, 68, 73, 75, 76, 79, 80, 82, 89, 91, 96, 97, 98, 99, 104, 106); twenty-one (54%) with minor concerns (58, 59, 60, 64, 69, 70, 71, 72, 74, 78, 83, 85, 86, 87, 88, 90, 94, 95, 102, 105, 107), and one (3%) with moderate concerns (63). | No or very minor concerns. The finding was well supported across the studies. Any variation between studies was minor and could be explained by contextual differences rather than reflecting contradictory evidence. | No or very minor concerns. A very large number of studies contributed to the sub-theme, representing various contexts and settings, with several providing nuanced, conceptually-rich insights. | Minor concerns. Eighteen studies (46%) included a partially relevant population, although most of these raised only minor concerns. Specifically, twelve studies still focused exclusively on sexual violence survivors (58, 64, 68, 69, 83, 87, 89, 90, 96, 98, 99, 105), and four examined intimate partner violence (63, 94, 95, 104), which remains highly relevant to the adulthood context of this review. Two studies included mixed samples that were not specifically violence-focused (79, 85). Eight studies (21%) did not focus on healthcare settings (67, 75, 82, 89, 94, 96, 102, 105). Four studies (89, 94, 96, 105) raised relevance concerns on both dimensions (i.e., they addressed neither healthcare settings nor adulthood sexual violence). | High confidence | Most studies (38 or 97%) had no or minor methodological concerns. There were no or very minor concerns about coherence or adequacy. This sub-theme had the largest number of contributing studies (39, or 78% of all included studies). The majority (33 or 85%) were directly relevant to the review question or raised only minor relevance concerns. The finding was well supported across the most conceptually rich studies and diverse healthcare settings. |
