# Supplementary material for: Trauma-informed healthcare from the perspectives of women who have experienced sexual violence in adulthood: a systematic review and meta-ethnography
Source: BMC Health Serv Res. 2025 Nov 27;26:13. doi: 10.1186/s12913-025-13584-x (PMC12763930; doi:10.1186/s12913-025-13584-x)
Supplement: Supplementary file 5 — Supplementary Material 5 [file 12913_2025_13584_MOESM5_ESM.docx]

Appendix E. Example of Translation Table

| **Key findings, concepts and themes** | **Translations** | **Example of third-order construct** |
| --- | --- | --- |
| Survivors described passively accepting care, feeling unsafe and unable to question healthcare provider authority.  Survivors described not feeling safe but continuing with procedures anyway.  Consent processes were described as unclear or absent e.g.,  parents signing consent forms without survivors' involvement.  The medical forensic exam was framed as something to tolerate rather than choose.  Some women wished they had refused parts of the forensic exam, and framed the experience as re-traumatising or re-victimising. | Agency was undermined by power imbalances, assumptions of compliance, and medical authority  De-prioritising or disregarding informed consent led to violation and harm  Care was framed as a re-victimisation when survivors felt unable or unsafe to voice preferences and needs | **A second attack.** Survivors described their agency as being eroded by power imbalances and assumptions of compliance with healthcare advice, requests, and expectations, rooted in medical authority and de-prioritisation of informed consent. These dynamics mirrored the silencing inherent in sexual violence, leaving survivors feeling unsafe, unable to express discomfort, articulate preferences, or exercise choice. In this way, healthcare settings could reproduce feelings of violation and powerlessness reminiscent of past experiences of sexual violence. |
